# Lysyl oxidase expression is associated with inferior outcome and Extramedullary disease of acute myeloid leukemia

**DOI:** 10.1186/s40364-020-00200-9

**Published:** 2020-06-12

**Authors:** Desiree Kunadt, Michael Kramer, Claudia Dill, Heidi Altmann, Lisa Wagenführ, Brigitte Mohr, Christian Thiede, Christoph Röllig, Johannes Schetelig, Martin Bornhäuser, Markus Schaich, Friedrich Stölzel

**Affiliations:** 1 Department of Internal Medicine I, University Hospital Carl Gustav Carus, Technical University of Dresden, Fetscherstrasse 74, 01307 Dresden, Germany; 2 Department of hematology, oncology and palliative care, Rems-Murr-Klinikum, Winnenden, Germany

**Keywords:** Acute myeloid leukemia, Lysyl oxidase, Extramedullary AML, Survival, Prognosis

## Abstract

**Background:**

Lysyl oxidase (LOX) has been described as necessary for premetastatic niche formation in epithelium-derived malignancies and its expression level therefore correlates with risk of metastatic disease and overall survival. However, its role in acute myeloid leukemia (AML) has not been sufficiently analyzed.

**Methods:**

We investigated LOX plasma expression in 683 AML patients (age 17–60 years) treated within the prospective AML2003 trial (NCT00180102). The optimal cut-off LOX value was determined using a minimal-*p*-value method dichotomizing patients into a LOX-high group (> 109 ng/mL, *n* = 272, 40%) and a LOX-low group (≤ 109 ng/mL, *n* = 411, 60%).

**Results:**

Higher LOX expression was associated with lower peripheral white blood cells, lower serum LDH, and a lower frequency of *FLT3*-ITD and *NPM1* mutations at diagnosis. Higher LOX expression was found significantly more frequently in patients with secondary AML and therapy-related AML, in patients with French-American-British M5 subtypes, and in patients with adverse-risk cytogenetics. Comparing patients in the LOX-high group and the LOX-low group revealed a 3-year overall survival (OS) of 47 and 53% (*p* = 0.022) and 3-year event-free survival (EFS) of 27 and 35% (*p* = 0.005), respectively. In the LOX-high group significantly more patients had extramedullary AML compared to the LOX-low group (*p* = 0.037). Combining extramedullary AML and LOX as interacting factors in a multivariate analysis resulted in an independent impact on survival for the LOX-high-extramedullary interaction for OS (HR = 2.25, *p* = 0.025) and EFS (HR = 2.48, *p* = 0.008). Furthermore, in patients with extramedullary disease (*n* = 59) the LOX level predicted survival. Patients within the LOX-low group had an OS of 43% and EFS of 36% as compared to the LOX-high group with an OS of 13% and EFS of 6% (*p* = 0.002 and *p* = 0.008, respectively).

**Conclusion:**

We hypothesize LOX expression to be a new potential biomarker to predict outcome in AML, specifically in AML subgroups such as the prognostic heterogeneous group of AML patients with extramedullary disease.

**Trial registration:**

This retrospective study was performed with patient samples registered within the prospective AML2003 trial (NCT00180102). Patients were enrolled between December 2003 and November 2009.

## Introduction

Within the last years various studies were able to demonstrate that in epithelium-derived cancers the occurrence of metastases is an orchestrated process involving hypoxia-induced mechanisms, neo-angiogenesis, recruitment of bone marrow-derived cells, and enzymatic deposition of extracellular matrices which result from certain genetic and epigenetic features of the underlying tumor cells themselves [1–4]. The consecutive formation of a so called premetastatic niche has been subject of studies which showed that abrogation of this premetastatic niche is able to eliminate the metastatic process itself in animal models [5].

Interestingly, these mechanisms have only been described for epithelial tumors so far. However, there is an increasing body of evidence that extramedullary manifestation of acute myeloid leukemia (AML) is often underreported at initial diagnosis and during relapse [6]. Studies have reported differences in frequency of clinically apparent extramedullary manifestations in adult AML between the time of diagnosis (approximately 9%) compared to relapse after allogeneic hematopoietic stem cell transplantation (HSCT) (5–12%) and relapsed AML after donor lymphocyte infusion or haploidentical HSCT (up to 32%) [7–12]. In a prospective trial using total body 18FDG-PET/CT imaging we could determine a 19% prevalence of metabolically active extramedullary AML at diagnosis with 60% of histologically confirmed extramedullary sites still being positive in follow-up imaging after intensive AML therapy [13].

While it is speculated that the true prevalence has yet to be defined, some reports have associated extramedullary AML with certain clinical and morphological features such as high white blood cell (WBC) count and French-American-British (FAB) M4/M5 subtypes [14–16] as well as distinct cytogenetic features such as 11q23 abnormalities, monosomy of chromosome 7, and abnormalities of chromosome 8 (most often trisomy 8) [15–19]. Published data show a high prevalence of extramedullary AML in *nucleophosmin 1* (*NPM1*) mutated AML patients as well as mutated *NPM1* in biopsied extramedullary sites in a high fraction of patients with extramedullary AML [20, 21]. In the subset of normal karyotype AML with *NPM1* mutations Garzon et al. were able to demonstrate that a significant proportion of patients have an up-regulation of microRNA 10a (miR-10a) in their leukemic cells [22]. While we were able to confirm these data, we demonstrated further that a significant proportion of these patients have extramedullary involvement at the time of diagnosis. MiRNA microarray data of these AML patients suggested an expression pattern of miRNAs that was also described in regulation processes for the initiation and the maintenance of a pre-metastatic niche. Furthermore, miR10a has a putative binding site to the 3’UTR of lysyl oxidase (LOX) which in return led to this work [23].

LOX is a copper-dependent, extracellularly secreted amine-oxidase mapped on human chromosome 5q23 that has been extensively described to be involved in the initiation of a premetastatic niche in epithelium-derived malignancies and in bone marrow fibrosis as well as in the development of pediatric acute megakaryoblastic leukemia [5, 24, 25]. Dawes et al. were able to increase LOX expression in the Molm14 AML cell line due to hypoxia exposure [26]. LOX expression is increased in tumor cells through hypoxia-inducible factor-1 (HIF-1) stimulation [27, 28]. After secretion of the LOX pro-enzyme into the extracellular space, proteolytic cleavage which is mainly catalyzed by bone morphogenic protein-1 (BMP-1) results in an active 30 kDa enzyme. LOX is thought to mainly catalyze deamination of peptidyl lysine residues in collagen and elastin molecules. Next, these resulting aldehydes undergo spontaneous reactions with other LOX-derived aldehydes or lysine residues. This results in cross-linking of collagen and elastin, which is essential for the stability of collagen fibers and integrity and elasticity of elastin fibers. It has been shown that LOX-dependent crosslinking of collagen fibers is involved in creating a “growth-permissive fibrotic microenvironment” for metastatic growth and the opportunity of tumor cell persistence [29]. Therefore, LOX expression levels correlate with distant metastasis-free and overall survival (OS) in various epithelium-derived cancers [5, 30]. Furthermore, animal models have demonstrated that different approaches inhibiting LOX have been able to sufficiently eliminate metastasis [5].

To date, little is known about the clinical implication of LOX expression in AML. In this study we investigated the plasma LOX expression from AML patients included in the AML2003 trial at diagnosis and correlated the results with clinical features and outcome of these patients.

## Material and methods

All available peripheral blood lithium-heparin plasma samples of 683 patients with AML (age 17–60 years) were analyzed for LOX expression. Lithium-heparin plasma samples were collected at initial diagnosis whenever possible. All patients were treated within the prospective, randomized, and multicentric AML2003 trial (NCT00180102). The study had been approved by the institutional review board (IRB) of the University of Dresden and all IRBs of participating centers of the Study Alliance Leukemia (SAL). The protocol was in agreement with the Helsinki Declaration. Written informed consent was obtained from each patient before inclusion. The risk-adapted design and inclusion criteria of the AML2003 trial have been described previously [31]. Briefly, patients were randomly assigned to receive either three cycles of high-dose cytarabine (HD-AraC) or MAC/MAMAC/MAC (mitoxantrone, amsacrine and high-dose cytarabine) as chemo-consolidation in a first step and in a second step to receive either an intensified, risk adapted and priority based consolidation strategy including early allogeneic hematopoietic stem cell transplantation (HSCT) within induction therapy-induced aplasia and autologous HSCT as second consolidation therapy or a standard consolidation strategy with allogeneic HSCT as first consolidation therapy resulting in four treatment arms. In total, 1179 patients between 16 and 60 years with newly diagnosed AML (excluding acute promyelocytic AML) or with myelodysplastic syndrome with excess blasts > 9% (MDS-EB2) were randomized at diagnosis and included in the AML2003 trial. Eligibility was based on confirmation of AML or MDS-EB2 by central morphologic and immunophenotyping analysis.

Bone marrow samples were processed in reference laboratories of the SAL study group. Cytogenetic analyses were performed using standard G-banding techniques and karyotyping according to the International System for Human Cytogenetic Nomenclature [32]. Fluorescent in situ hybridization (FISH) techniques were performed according to the manufacturer’s recommendations. Molecular analyses for mutations of *FLT3-*ITD and *NPM1* mutations were performed as previously published in detail [33, 34]. Extramedullary AML was defined according to the World Health Organization (WHO) 2008 criteria requiring a tissue sample from an extramedullary site (i.e. anatomical site other than the bone marrow) composed of myeloid blasts destroying the tissue architecture [35]. Data was collected and certified by the SAL Data Center.

Peripheral blood lithium-heparin plasma samples were analyzed for LOX concentration using an enzymatic assay (Amplite Fluorimetric LOX Assay Kit. AAT Bioquest, Sunnyvale, CA, USA) according to the manufacturer’s instructions [36]. All fluorescence reads were performed in triplicate with recombinant human LOXL2 (R&D Systems, Minneapolis, MN, USA) for standard curve estimation. Signals were read by a fluorescence microplate reader at Ex/Em 540/590 nm (Berthold Technologies, Bald Wildbad, Germany). Supernatant of the *NPM1* mutated AML cell line OCI/AML3 served as internal control and for analysis of a day-to-day variability. OCI/AML3 cells were purchased from the Leibnitz-Institute – German Collection of Microorganisms and Cell Cultures (DSMZ, Braunschweig, Germany).

## Statistical analysis

Overall survival (OS) and event-free survival (EFS) were measured from the date of entry into the study to the date of event or the last follow up. Death, induction failure, and relapse were considered events for EFS. Complete remission (CR) was defined according to standard criteria [37]. The range of LOX serum concentration for all patients was 0–2184 ng/mL with a mean LOX concentration of 119.7 ng/mL. Inspection of the Martingale residuals of a Cox model testing the influence of LOX as a continuous variable on OS revealed that a cut-off model might be most appropriate. The optimal cut-off LOX value was determined using a minimal-*p*-value method [38]. The resulting logarithmic logLOX value = 2.0403 (109 ng/mL) was used to dichotomize all patients into a LOX-high group (> 109 ng/mL, *n* = 272, 40%) and a LOX-low group (≤ 109 ng/mL, *n* = 411, 60%). Differences of continuous variables between both groups were analysed by means of the Mann-Whitney-U-Test. Differences of the distribution of categorical variables were analysed using the uncorrected Chi-Squared-Test. The method of Kaplan-Meier was used to estimate OS and EFS. Survival distributions were compared using the log-rank test. Prognostic parameters were tested in a Cox regression model for OS and EFS. Cytogenetic karyotype, *FLT3*-ITD mutation status, *NPM1* mutation status, extramedullary AML, and LOX expression at diagnosis were used as dichotomized variables whereas age, and logarithmic (to base 10) WBC at diagnosis, and the bone marrow blast count at day 15 after induction chemotherapy were used as continuous variables. All statistical analyses were performed using SPSS version 19.0 (SPSS, Chicago, IL, USA) and R 2.15.1.

## Results

### LOX expression and patient characteristics

For internal validation we first identified the OCI/AML3 cell line as representing a cell line with a high LOX expression in the supernatant. We performed independent supernatant day-to-day variability measurements in triplicate on each day on three consecutive days resulting in a mean LOX concentration of 792.5 ng/mL and a standard deviation of 101.9 ng/mL with a day-to-day variability of 13%.

As mentioned, a logarithmic logLOX value was identified which dichotomizes the analyzed 683 patients in a LOX-low group comprised of 411 patients (60%) and a LOX-high group with 272 patients (40%). Detailed patient characteristics are depicted in Table 1. Comparing clinical and laboratory baseline characteristics at initial diagnosis AML patients in the LOX-high group exposed significantly less bone marrow blasts (54% vs. 63%, *p* < 0.001), lower WBC counts (6.5 GPt/L vs. 26 GPt/L, *p* < 0.001), and lower serum LDH (389 U/l vs. 5247 U/l, p < 0.001) compared to those patients with low LOX expression, respectively. Furthermore, a significantly lower proportion of *FLT3*-ITD mutations (17% vs. 30%, p < 0.001) and *NPM1* mutations (26% vs. 38%, *p* = 0.002) were detected. The LOX-high group consisted of significantly more patients with therapy-related AML (t-AML) (7% vs. 4%) and patients with a preceding MDS (mdsAML) (11% vs. 4%), *p* = 0.001, a higher proportion of monocytic morphology, more cytogenetically defined adverse-risk karyotypes (34% vs. 16%), and less normal karyotypes (58% vs. 70%) and favourable karyotypes (9% vs. 14%), *p* < 0.001. In detail, single cytogenetic abnormalities that were identified more frequently among the LOX-high group were complex karyotype (defined as ≥3 independent karyotypic abnormalities) (*p* = 0.005), del(5q) (*p* = 0.007), t (9;11) (*p* = 0.002), and other abnormalities of chromosome 11q23 (abnl (11q23)) (*p* < 0.001). Abnormalities belonging to a favorable or undefined risk category that were frequently higher expressed in the LOX-high group were monosomies of chromosomes other than chromosome 5 or 7 (*p* = 0.004), and trisomy of chromosome 8 (*p* = 0.001). AML with normal karyotype or inversion of chromosome 16 were less frequently observed in the LOX-high group, *p* < 0.001 and *p* = 0.022, respectively. As hypothesized initially, we could confirm a significantly higher proportion of patients with histologically confirmed extramedullary AML as defined per WHO criteria in the LOX-high group, *p* = 0.037.

**Table 1:** Study patients’ characteristics

	**all AML** ***n*** **= 683**	**LOX-low group** ***n*** **= 411, 60%**	**LOX-high group** ***n*** **= 272, 40%**	***p*-value**
age at diagnosis median (range)	46 (16–60)	47 (16–60)	50 (18–60)	0.045
Gender, Female, no. (percent)	364 (53)	226 (55)	138 (51)	0.276
Bone marrow blasts at diagnosis [percent] median (range)	60.5 (3–100)	63.25 (8–100)	54.25 (3–96)	< 0.001
WBC count at diagnosis [Gpt/L] median (range)	14.5 (0.3–353)	26.1 (0.3–353)	6.5 (0.6–243.4)	< 0.001
Platelet count at diagnosis [Gpt/L] median (range)	52 (4–1308)	51 (4–1308)	52 (4–793)	0.626
Serum LDH at diagnosis [U/l] median (range)	492 (100–7369)	527 (144–5934)	389 (100–7369)	< 0.001
*FLT3*-ITD mutational status positive, no. (percent)^a^	167 (25)	122 (30)	45 (17)	< 0.001
*NPM1* mutational status positive, no. (percent)^b^	226 (33)	155 (38)	71 (26)	0.002
Combined *NPM1/FLT3* status, no. (percent)				
*NPM1* wt/*FLT3* wt	377 (56)	204 (50)	173 (65)	< 0.001
*NPM1* wt/*FLT3* mut	70 (10)	46 (11)	24 (9)	
*NPM1* mut/*FLT3* wt	130 (19)	80 (20)	50 (19)	
*NPM1* mut/*FLT3* mut	96 (14)	75 (18)	21 (8)	
Disease status at diagnosis, no. (percent)				
De novo AML	603 (88)	378 (92)	225 (83)	0.001
t-AML	35 (5)	17 (4)	18 (7)	
mdsAML	45 (7)	16 (4)	29 (11)	
FAB subtypes at diagnosis, no. (percent)				
M0	27 (4)	11 (3)	16 (6)	0.001
M1	134 (20)	96 (23)	38 (14)	
M2	204 (30)	119 (29)	85 (31)	
M4	88 (13)	62 (15)	26 (10)	
M4eo	36 (5)	28 (7)	8 (3)	
M5a	71 (10)	35 (8)	36 (13)	
M5b	22 (3)	11 (3)	11 (4)	
M5 other	7 (1)	4 (1)	4 (1)	
M6	23 (3)	9 (2)	14 (5)	
M7	6 (1)	3 (1)	3 (1)	
RAEB	19 (3)	9 (2)	10 (4)	
RAEB-T	33 (5)	17 (4)	16 (6)	
Cytogenetic subgroups, no. (percent)				
adverse risk	157 (23)	65 (16)	92 (34)	< 0.001
intermediate risk	444 (65)	287 (70)	157 (58)	
favorable risk	82 (12)	59 (14)	23 (8)	
Common cytogenetic aberrations, no. (percent)				
Complex karyotype	90 (13)	42 (10)	48 (18)	0.005
Normal karyotype	347 (49)	242 (59)	95 (35)	< 0.001
t(8;21)	39 (6)	26 (6)	13 (5)	0.394
inv16	43 (6)	33 (8)	10 (4)	0.022
monosomy 5	7 (1)	3 (1)	4 (1)	0.347
del5q	48 (7)	20 (5)	28 (10)	0.007
monosomy 7	30 (4)	14 (3)	16 (6)	0.122
del7q	20 (3)	9 (2)	11 (4)	0.159
monosomy other than chromosomes 5 or 7	43 (6)	17 (4)	26 (10)	0.004
trisomy 8	74 (11)	31 (7)	43 (16)	0.001
t(9;11)	17 (2)	4 (1)	13 (5)	0.002
abnl(11q23)	59 (9)	22 (5)	37 (14)	< 0.001
Extramedullary manifestation of AML, no. (percent)	59 (9)	28 (7)	31 (11)	0.037

Table 1 The study patients’ characteristics at diagnosis are listed

*Abbreviations: AML* acute myeloid leukemia, *FAB* French American British classification of acute leukemia, *FLT3*-ITD, *FMS-like tyrosine kinase 3* internal tandem duplication, *LDH* lactate dehydrogenase, *MDS* myelodysplastic syndrome, *mut* mutated, *NPM1 Nucleophosmin 1*, *RAEB* refractory anemia with excess blasts, *t-AML* therapy-related AML, *mdsAML* AML with preceding MDS, *WBC* white blood count, *wt* wildtype

^a^*FLT3* mutation screening was performed in *n* = 678 patients at initial diagnosis

^b^*NPM1* mutation screening was performed in *n* = 674 patients at initial diagnosis

### Overall- and event-free survival

**Fig. 1:**
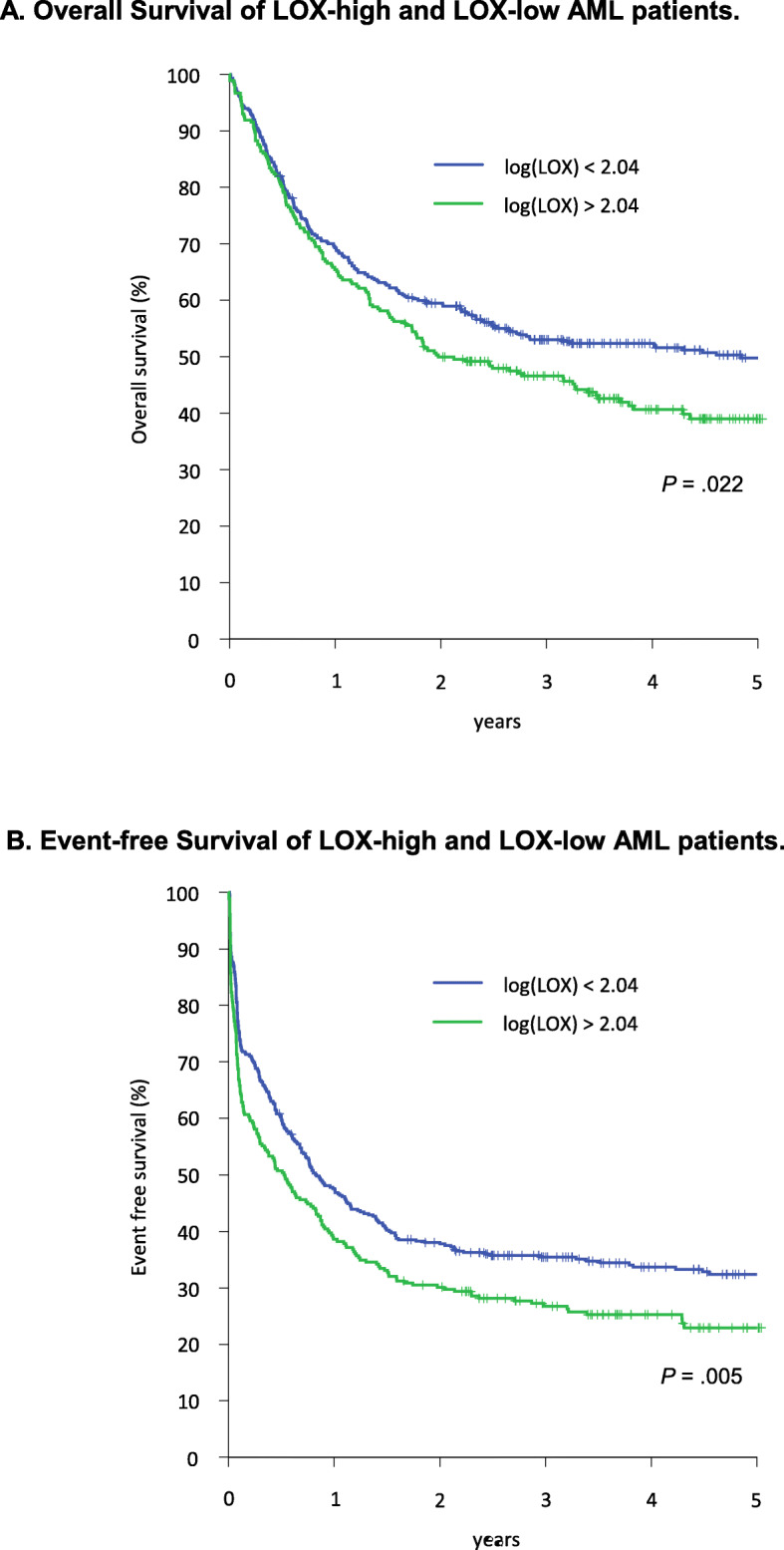
**a** and **b** Overall Survival (OS) (Fig. a) and Event-free Survival (EFS) of 683 AML patients dichotomized to the LOX-high (n = 272) and LOX-low (n = 411) group. LOX-high patients are denoted in green, LOX-low patients are denoted in blue color. The logarithmic logLOX value = 2.0403 (109 ng/mL) was used to dichotomize all patients in both groups, respectively. For LOX-high patients 3-year OS was 47% (95% CI, 40–53%) and for LOX-low patients 53% (95% CI, 48–58%), *p* = .022. Three-year EFS for LOX-high patients was 27% (95% CI, 21–32%) and for LOX-low patients 35% (95% CI, 30–40%), *p* = 0.005

Next we analyzed the impact of the LOX-high and LOX-low status on survival in these AML patients. Comparing LOX-high and LOX-low AML patients revealed a 3-year OS of 47% (95% CI 40–53%) and 53% (95% CI 48–58%, *p* = 0.022), and a 3-year EFS of 27% (95% CI 21–32%) and 35% (95% CI 30–40%, *p* = 0.005), respectively (Fig. 1a and b). Since the LOX-high group contained significantly more patients with histologically reported extramedullary AML compared to the LOX-low group, *p* = 0.037, we aimed to focus further on this subgroup. In order to analyze the impact of LOX expression on survival outcomes adjusting for potential confounding variables in a multivariate analysis, we performed a Cox regression analysis including the prognostic parameters karyotype, age, *FLT3*-ITD mutation, *NPM1* mutation, logarithmic WBC (to base 10), bone marrow blast count at day 15 after induction chemotherapy, extramedullary disease, and LOX expression. In the presence of these variables LOX expression lost its influence on OS and EFS. However, since extramedullary AML and LOX seem to be related on a biological level, we introduced an interaction term in the Cox model. We identified a robust impact of LOX expression and extramedullary manifestation on OS (HR = 2.25 (95% CI, 1.11–4.56), *p* = 0.025) and EFS (HR = 2.48 (95% CI 1.26–4.86), *p* = 0.008) (Table 2). As previously shown, different forms of consolidation (either 3 cycles of HD-AraC or MAC/MAMAC/MAC as part of the AML2003 trial) did not lead to significant differences in outcome [31].

**Table 2:** Multivariable analysis concerning overall and event-free survival

Factor	**Overall Survival**		**Event-free Survival**	
	**HR** **[95% CI]**	***p*-value**	**HR** **[95% CI]**	***p*-value**
Low Risk Karyotype	0.486 [0.287–0.825]	0.007	0.553 [0.374–0.818]	0.003
High Risk Karyotype	1.858 [1.404–2.459]	< 0.001	1.407 [1.088–1.818]	0.009
Higher Age^a^	1.034 [1.021–1.047]	< 0.001	1.021 [1.011–1.032]	< 0.001
*FLT3*-ITD positivity	1.834 [1.376–2.445]	< 0.001	1.430 [1.109–1.843]	0.006
*NPM1* mutation	0.527 [0.385–0.723]	< 0.001	0.530 [0.408–0.689]	< 0.001
Logarithmic (to base 10) WBC	1.191 [0.962–1.474]	0.109	1.030 [0.857–1.237]	0.754
Bone Marrow Blast Count at Day 15 after Induction Chemotherapy^b^	1.010 [1.005–1.014]	< 0.001	1.028 [1.023–1.033]	< 0.001
Extramedullary Disease	1.203 [0.698–2.076]	0.506	0.888 [0.535–1.476]	0.648
high LOX Expression	0.956 [0.711–1.285]	0.766	1.034 [0.804–1.329]	0.795
Interaction of Extramedullary Disease and high LOX Expression	2.246 [1.106–4.559]	0.025	2.478 [1.264–4.857]	0.008

Table 2 Results of multivariate testing for overall survival and event-free survival including HR, 95% CI and *p*-values

*Abbreviations: HR* hazard ratio, 95% *CIs* 95% confidence interval

^a^age as a continuous variable in years

^b^bone marrow blast count as a continuous variable in percent

### Subgroup analysis in extramedullary AML

**Fig. 2:**
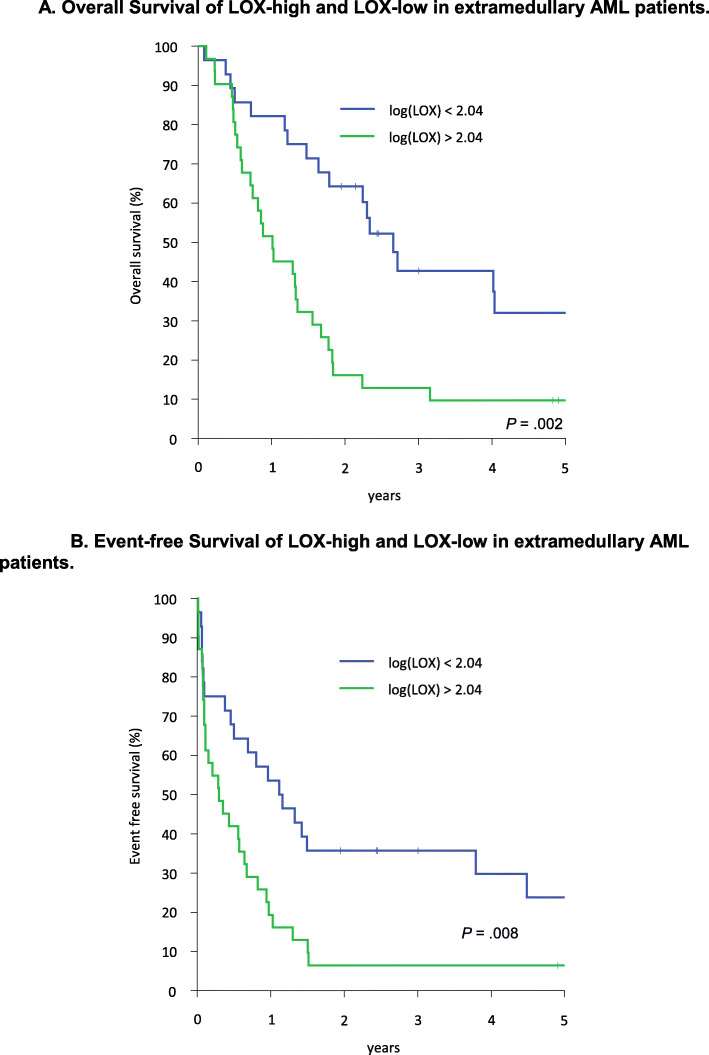
**a** and **b** Overall Survival (OS) (Fig. 1a) and Event-free Survival (EFS) of 59 AML patients with extramedullary disease dichotomized to the LOX-high (*n* = 31) and LOX-low (*n* = 28) group. LOX-high patients are denoted in green, LOX-low patients are denoted in blue color. The logarithmic logLOX value = 2.0403 (109 ng/mL) was used to dichotomize all patients in both groups, respectively. For LOX-high patients 3-year OS was 13% (95% CI, 1–25%) and for LOX-low patients 43% (95% CI, 23–63%), *p* = .002. Three-year EFS for LOX-high patients was 6% (95% CI, 0–15%) and for LOX-low patients 36% (95% CI, 17–54%), *p* = 0.008

Since we initially hypothesized that a higher proportion of patients with extramedullary AML would have elevated high LOX plasma levels at diagnosis, we analyzed the cohort of patients with reported and histologically confirmed extramedullary AML at initial diagnosis concurrently with AML without extramedullary leukemic manifestations. In total 59 AML patients with histologically confirmed extramedullary AML at diagnosis and available peripheral blood samples from initial diagnosis could be analyzed for LOX expression. LOX high (*n* = 31) and LOX low patients (*n* = 28) were about equally distributed. The clinically and laboratory baseline characteristics of the 59 patients are depicted in Table 3. Interestingly, in this comparison of LOX-high (*n* = 31) and LOX-low (n = 28) patients, only a higher proportion of t-AML and MDS-related AML (*p* = 0.049), higher numbers of monocytic AML according to FAB-subtype (*p* = 0.003), and more patients with cytogenetically defined high-risk abnormalities (*p* = 0.001) remained as statistically significant different in these groups in univariate analysis. Regarding single cytogenetic abnormalities we again observed a two-fold higher rate of complex karyotypes and trisomy 8 in the LOX-high cohort (*n* = 8, 26% vs. *n* = 3, 11%, *p* = 0.137). For abnl (11q23) however, a more pronounced significant difference between LOX-high and LOX-low patients was observed despite of low patient numbers in this cohort (*n* = 9, 29% vs. *n* = 1, 4%, *p* = 0.009), respectively. Furthermore, also in patients with extramedullary AML the LOX expression predicted survival. Patients within the LOX-low group had an OS of 43% (95% CI, 23–63%) and EFS of 36% (95% CI, 17–54%) as compared to the LOX-high group with an OS of 13% (95% CI, 1–25%) and EFS of 6% (95% CI, 0–15%), *p* = 0.002 and *p* = 0.008, respectively, in univariate analysis (Fig. 2a and b).

**Table 3:** Characteristics of LOX-low/LOX-high extramedullary AML

	**all EM AML** ***n*** **= 59**			
	**all EM AML** ***n*** **= 59**	**LOX-low group** ***n*** **= 28, 60%**	**LOX-high group** ***n*** **= 31, 40%**	***p*-value**
age at diagnosis median (range)	46 (17–60)	45 (17–60)	49 (22–59)	0.538
Gender, Female, no. (percent)	24 (41)	13 (46)	11 (35)	0.393
Bone marrow blasts at diagnosis [percent] median (range)	63 (20–96)	68.5 (20–94)	62.5 (20.5–96)	0.792
WBC count at diagnosis [Gpt/L] median (range)	26.9 (1.3–191.4)	38 (1.4–191.4)	18.8 (1.3–189.1)	0.370
Platelet count at diagnosis [Gpt/L] median (range)	52 (13–357)	61 (13–231)	50 (13–357)	0.970
Serum LDH at diagnosis [U/l] median (range)	707 (145–4945)	694 (186–4945)	768 (145–3973)	0.439
*FLT3*-ITD mutational status, positive, no. (percent)	12 (25)	8 (29)	4 (13)	0.135
*NPM1* mutational status, *NPM1* mut, no. (percent)	21 (36)	10 (37)	11 (35)	0.902
Combined *NPM1/FLT3* status, no. (percent)				
*NPM1* wt/*FLT3* wt	32 (55)	14 (52)	18 (58)	0.413
*NPM1* wt/*FLT3* mut	5 (9)	3 (11)	2 (6)	
*NPM1* mut/*FLT3* wt	14 (24)	5 (18)	9 (29)	
*NPM1* mut/*FLT3* mut	7 (12)	5 (18)	2 (6)	
Disease status at diagnosis, no. (percent)				
De novo AML	53 (90)	28 (100)	25 (81)	0.049
tAML	5 (8)	0 (0)	5 (16)	
mdsAML	1 (2)	0 (0)	1 (3)	
FAB subtypes at diagnosis, no. (percent)				
M0	2 (3)	0 (0)	2 (6)	0.003
M1	11 (19)	9 (32)	2 (6)	
M2	14 (24)	10 (36)	4 (13)	
M4	9 (15)	2 (7)	7 (23)	
M4eo	2 (3)	2 (7)	0 (0)	
M5a	11 (19)	1 (4)	10 (32)	
M5b	2 (3)	0 (0)	2 (6)	
M6	2 (3)	1 (4)	1 (3)	
M7	1 (2)	0 (0)	1 (3)	
RAEB-T	3 (5)	1 (4)	2 (6)	
M5 other	2 (3)	2 (7)	0 (0)	
Cytogenetic subgroups, no. (percent)				
High risk	19 (32)	3 (11)	16 (52)	0.001
Standard risk	33 (56)	19 (68)	14 (45)	
Low risk	7 (12)	6 (21)	1 (3)	
Detailed karyotypes, no. (percent)				
Complex karyotype	12 (20)	3 (11)	9 (29)	0.081
Normal karyotype	18 (32)	12 (44)	6 (20)	0.047
t(8;21)	4 (7)	3 (11)	1 (3)	0.253
inv16	3 (5)	3 (11)	0 (0)	0.061
monosomy 5	2 (3)	0 (0)	2 (6)	0.171
del5q	3 (5)	1 (4)	2 (6)	0.615
monosomy 7	1 (2)	1 (4)	0 (0)	0.289
del7q	3 (5)	1 (4)	2 (6)	0.615
monosomy other than chromosomes 5 or 7	3 (5)	1 (4)	2 (6)	0.615
trisomy 8	11 (19)	3 (11)	8 (26)	0.137
t(9;11)	3 (5)	0 (0)	3 (10)	0.091
abnl(11q23)	10 (17)	1 (4)	9 (29)	0.009

Table 3 Univariate results of EM-AML patients’ characteristics, divided in LOX-low and LOX-high group

*Abbreviations: AML* acute myeloid leukemia, *FAB* French American British classification of acute leukemia, *FLT3*-ITD *FMS-like tyrosine kinase 3* internal tandem duplication, *LDH* lactate dehydrogenase, *MDS* myelodysplastic syndrome, *NPM*1 *Nucleophosmin 1*, *RAEB* refractory anemia with excess blasts, *t-AML* therapy-related AML, *mdsAML* AML with preceding MDS, *WBC* white blood count

## Discussion

In the current analysis we observed that high LOX expression correlated with the occurrence of extramedullary AML and certain AML features which occur at a high frequency with extramedullary AML. Furthermore, high LOX expression in patients’ plasma was found to be associated with statistically significant worse OS and EFS in AML patients under the age of 60 years in a univariate setting – a finding which we found to be even more pronounced in the patient group with reported extramedullary AML. Though, one must speculate that due to the lack of routine screening for extramedullary AML at diagnosis, the prevalence of EM AML might be higher than reported (Stölzel F et al., The prevalence of extramedullary acute myeloid leukemia detected by 18FDG-PET/CT: final results from the Prospective PETAML Trial. Haematologica. 2019 Aug 29. pii: haematol.2019.223032). Consequently, we suggest a considerable number of unknown EM AML patients currently still classified as “normal” AML without clinical apparent extramedullary manifestations at diagnosis for which LOX could also be a potential marker for prognosis.

The multivariate analyses revealed that this association is mainly caused by a higher incidence of adverse cytogenetic aberrations and myelodysplastic features and a lower incidence of favorable cytogenetics and *NPM1* mutations.

Furthermore, the statistically significant interaction between higher LOX expression and extramedullary AML suggests a potentially pathophysiological relevant mechanism involved in extramedullary homing and growth of AML and may offer further insights into AML biology. The prognostic heterogeneous group of AML patients with extramedullary disease can be separated in those with superior and those with inferior survival by applying the peripheral blood lithium-heparin plasma LOX expression level at diagnosis. This might be of importance for future applications in clinical as well as in scientific settings since so far, the survival of AML patients with extramedullary disease at diagnosis has been discussed controversially. Importantly, our data need to be confirmed in an independent validation patient cohort which might prove difficult since most large AML trials do not collect and bank plasma samples. Further experimental studies are needed to address the functional modalities of how LOX is regulated and how it contributes to migratory and adhesion properties in AML. Possible caveats for future studies addressing these questions might be that few AML trials include the systematic screening, documentation and analysis of extramedullary AML - a phenomenon that already led to an underreporting of extramedullary AML after allogeneic HSCT [39]. Even more so, data exist demonstrating that allogeneic HSCT performed as second allogeneic transplantation after relapsed disease or in a haploidentical setting might be associated with a higher prevalence of extramedullary AML at relapse, implicating clonal evolution and immune-escape phenomena involved as well as varying susceptibilities for AML seeds in different tissues [40]. In clinical reality, only those extramedullary AML manifestations which are clinically apparent or identified by imaging studies [41, 42] and which are histologically confirmed are taken into account in scientific analyses like ours [11, 13, 40–42]. This could theoretically translate into an underreporting of extramedullary AML in general. One approach that has been proposed in this context is the detection of *WT1* expression by quantitative PCR which was reported to predict extramedullary AML. Patients with extramedullary relapse had high expression levels in the peripheral blood whereas the *WT1* level in the bone marrow remained negative [43]. Furthermore, *AML1-MTG8* expression levels were shown to possibly predict EM relapse, specifically in t (8;21) AML [44].

Further indicators linking LOX to extramedullary manifestation of AML in our report is certain characteristics known to be more common in extramedullary AML were more frequently observed in the LOX-high group, i.e. higher numbers of patients with monocytic morphology and certain karyotypic abnormalities that have been associated with extramedullary AML such as e.g. complex aberrant karyotypes, trisomy of chromosome 8, or 11q23 abnormalities amongst others [8, 17, 19, 20, 45, 46]. Worth considering for future studies and analyses on the pathophysiology and the genetic evolution of extramedullary AML is the so far unacquainted matter that karyotypic features of AML clones residing in the bone marrow might be different from those residing in extramedullary sites as demonstrated by array-CGH technology [18].

However, without the existence of a homogenous tumor population within one individual it was speculated by Fidler et al. that in epithelial cancers rare subpopulations of malignant clones exist which are endowed with some or several “metastasis-promoting functions” [47]. Furthermore, it was hypothesized that highly metastatic clones from a heterogenous tumor cell population have a higher rate of genetic variation and a higher rate of mutations than non-metastatic clones from the same tumor cell population [48]. This concurs with the observation of a higher incidence of clonal heterogeneity in extramedullary AML (i.e. complex karyotypes) [18]. Alas, studies performing comparative genetic analyses in extramedullary AML samples and bone marrow or blood samples specifically are needed in order to exploit the different functional implications of different AML clones. One aspect of the leukemia cell invadosome in extramedullary infiltration has been studied revealing that interactions between matrix-metalloproteinase-9 and leukocyte β2-Integrin are necessary for migration, for progression, and chemosensitivity in AML [49, 50].

While the three-dimensional structure of the human LOX protein has not been resolved so far, it furthermore needs to be explored which isoforms of LOX are relevant in the context of AML and whether its regulation through HIFs in hypoxia, its ability to recruit CD11b myeloid cells which adhere to crosslinked collagen and produce matrix-metalloproteinase-2 enhancing premetastatic niche formation is of relevance in AML. Also the ability of LOX to increase vascular endothelial growth factor (VEGF) expression and secretion which promotes angiogenesis needs to be reflected in this context [51]. Finally, follow-up plasma samples during treatment and determination of LOX activity could reflect a response to treatment through decrease in LOX activity as one might hypothesize in AML and in patients with extramedullary AML, specifically.

## Conclusion

The correlation between high LOX expression and extramedullary AML suggests a potentially relevant mechanism involved in extramedullary adhesion, migration, and growth of AML and may offer further insights into AML homing biology and therapeutic targeting options. However, one must speculate that due to the lack of routine screening for extramedullary AML at diagnosis, there might be a considerable amount of unknown extramedullary AML at diagnosis, especially in cases without clinical apparent extramedullary manifestations, for which LOX could also be a potential marker for prognosis. A subsequent approach in order to modify or even selectively inhibit LOX might be reasonable for an attempt to improve the clinically inferior and still dissatisfying outcome of these patients.

## Data Availability

The datasets generated and analyzed during the study are available from the corresponding author on reasonable request.

## Abbreviations

3’UTR: 3′ untranslated region
AML: Acute myeloid leukemia
BMP-1: Bone morphogenic protein-1
CR: Complete remission
EFS: Event-free survival
FAB: French-American-British
FISH: Fluorescent in situ hybridization
FLT3-ITD: FMS-like tyrosine kinase 3 internal tandem duplication
HIF-1: Hypoxia-inducible factor-1
HSCT: Hematopoietic stem cell transplantation
IRB: Institutional review board
LDH: Lactate dehydrogenase
LOX: Lysyl oxidase
MAC/MAMAC/MAC: Mitoxantrone, amsacrine, high-dose cytarabine
mdsAML: AML with preceding MDS
MDS-EB2: Myelodysplastic syndrome with excess blasts > 9%
miR-10a: microRNA 10a
NPM1: Nucleophosmin 1
OS: Overall Survival
SAL: Study Alliance Leukemia
t-AML: therapy-related AML
VEGF: vascular endothelial growth factor
WBC: white blood count
WHO: World Health Organization
WT1: Wilms’ tumor protein 1
